# Sperm mixing in the polyandrous leaf-cutting ant *Acromyrmex echinatior*

**DOI:** 10.1002/ece3.1176

**Published:** 2014-09-02

**Authors:** Marlene Stürup, David R Nash, William O H Hughes, Jacobus J Boomsma

**Affiliations:** 1 Department of Biology, Centre for Social Evolution, University of CopenhagenUniversitetsparken 15, Copenhagen, 2100, Denmark; 2 School of Life Sciences, University of SussexBrighton, BN1 9QG, UK

**Keywords:** Genetic diversity, patrilines, polyandry, sperm clumping, sperm mixing

## Abstract

The insemination of queens by sperm from multiple males (polyandry) has evolved in a number of eusocial insect lineages despite the likely costs of the behavior. The selective advantages in terms of colony fitness must therefore also be significant and there is now good evidence that polyandry increases genetic variation among workers, thereby improving the efficiency of division of labor, resistance against disease, and diluting the impact of genetically incompatible matings. However, these advantages will only be maximized if the sperm of initially discrete ejaculates are mixed when stored in queen spermathecae and used for egg fertilization in a “fair raffle.” Remarkably, however, very few studies have addressed the level of sperm mixing in social insects. Here we analyzed sperm use over time in the highly polyandrous leaf-cutting ant *Acromyrmex echinatior*. We genotyped cohorts of workers produced either 2 months apart or up to over a year apart, and batches of eggs laid up to over 2 years apart, and tested whether fluctuations in patriline distributions deviated from random. We show that the representation of father males in both egg and worker cohorts does not change over time, consistent with obligatorily polyandrous queens maximizing their fitness when workers are as genetically diverse as possible.

## Introduction

The evolution of polyandry (insemination of queens with sperm from multiple males) is an important problem in evolutionary biology because the behavior imposes significant costs to females in terms of increased energy expenditure, predation, and disease transmission (Arnqvist and Nilsson 2000; Crozier and Fjerdingstad 2001; Simmons 2005). Polyandry has nonetheless evolved in eusocial insects that often have demanding mating flights, reaching very high and apparently obligate levels in multiple genera (Boomsma and Ratnieks 1996; Strassmann 2001; Kronauer et al. 2004; Hughes et al. 2008; Baer 2010). Although this initially appeared contrary to understanding the evolution of eusociality as being driven by kin selection (Page and Metcalf 1982; Crozier and Page 1985; Boomsma and Ratnieks 1996), multiple queen mating is now known to be an evolutionarily derived trait that evolved only after workers had permanently lost reproductive totipotency (i.e., the ability to mate and reproduce sexually; Boomsma 2007; Hughes et al. 2008), consistent with an original suggestion by Hamilton (1964).

While many hypotheses for the evolution of polyandry have been suggested (Boomsma and Ratnieks 1996; Crozier and Fjerdingstad 2001), the best supported explanations are that high levels of multiple mating by queens result in fitter colonies because multiple paternity increases the genetic variability of workers within a colony. If individuals vary genotypically in their susceptibility to parasites, polyandrous colonies should be less vulnerable to infections (Hamilton 1987; Schmid-Hempel 1998; Boomsma et al. 2005b). Such positive effects have now been documented for a number of eusocial insects that independently evolved permanently unmated worker castes (Baer and Schmid-Hempel 1999; Hughes et al. 2002; Tarpy 2003; Hughes and Boomsma 2004; Johnson et al. 2013). Particularly in eusocial lineages with perennial colonies, sophisticated division of labor is a key element contributing to ecological success (Oster and Wilson 1978; Hölldobler and Wilson 1990), and polyandrous colonies can achieve more optimal task allocation when genotypes vary in their propensity to perform different tasks or to develop into different castes (Hughes et al. 2003; Jones et al. 2004; Jaffé et al. 2007; Mattila and Seeley 2007; Oldroyd and Fewell 2007; Smith et al. 2008).

Although polyandry reduces worker relatedness and therefore the indirect fitness benefits of cooperation, this can no longer affect the extent of eusocial commitment when workers have irreversibly lost the ability to mate, initiate their own colonies, and live independently (Hughes et al. 2008; Boomsma 2013). This logic does not preclude that obligatorily eusocial workers in many species are still capable of laying haploid eggs that develop into males (Wilson 1971; Bourke 1988) and that other workers may raise such males even when worker reproduction reduces overall colony productivity (Wenseleers et al. 2004). Such selfish traits are always expected to evolve when relatedness ratios vary, and individuals possess power to pursue their own inclusive fitness interests rather than complying with the interests of their nestmates (Beekman and Ratnieks 2003).

Polyandry in eusocial hymenoptera is of fundamental interest because it reduces queen–worker conflict over both male production and sex allocation (Ratnieks et al. 2006). In colonies headed by a single monandrous queen, workers are more related to worker-produced males (0.375) than to queen-produced males (0.25), making them favor male production by their sister workers (Ratnieks 1988). By storing and using sperm from multiple males, a queen reduces the average relatedness of workers to males produced by their sisters and therefore subjects them to selection to favor her own sons, provided they can recognize them as such (Ratnieks 1995; Boomsma and d’Ettorre 2013). In addition, polyandry alters queen–worker conflict over the sex ratio of gynes (new virgin queens) to males produced. On relatedness grounds, the optimum sex ratio for an outbred queen is always 1:1, but relatedness asymmetry among daughters in haplodiploids means that the optimal sex allocation for workers is 3:1 when all colonies are headed by a monandrous queen (worker relatedness to sister gynes and brothers is 0.75 and 0.25, respectively). Polyandry brings the optimum sex ratio for workers closer to the queen’s and therefore reduces queen–worker conflict, although conflict reduction can only select for increased polyandry under very specific conditions (Ratnieks and Boomsma 1995). However, relatedness between colony sisters at any one time does not merely depend on the number of matings per se, but rather on how ejaculates are used to fertilize eggs.

It has long been recognized that ejaculates can vary in size and hence representation in eusocial hymenopteran offspring (Starr 1984; Pamilo 1993; Boomsma and Ratnieks 1996; Nielsen et al. 2003; Boomsma 2013), but the temporal dynamics of sperm allocation has remained understudied. If sperm would somehow remain clumped after storage and thus used sequentially over the life spans of queens, as originally hypothesized by Trivers and Hare (1976), worker cohorts would be more closely related than if sperm was mixed and used randomly. If workers could perceive such higher sister-relatedness and bias sex ratios toward more virgin queens rather than males, sperm clumping could benefit the reproductive interests of the father males because colonies would produce more gynes who carry father genes (Boomsma 1996). However, such split sex ratios have only been shown in ant species where polyandry is facultative (Boomsma and Sundström 1998) and not in all studies (Pamilo and Seppa 1994; Fjerdingstad et al. 2003).

Some of the best evidence for sperm mixing rather than clumping in the spermatheca, and subsequent random sperm use during egg fertilization comes from honeybees and army ants, where offspring paternity analyses have shown that, while sperm is initially somewhat clumped immediately following mating, it becomes completely mixed during the next few months (Laidlaw and Page 1984; Franck et al. 1999, 2002; Kronauer et al. 2006). This suggests that the original coherence of ejaculates is gradually lost during the early storage process in the spermatheca even though ejaculates were inseminated sequentially. Sperm clumping does, however, persist in at least one social insect species, the ant *Formica truncorum*, where multiple mating is facultative and workers split their sex allocation in response to the mating status of their mother (Sundström 1994; Boomsma et al. 2003). A recent study has also suggested that sperm clumping may occur in highly polyandrous *Pogonomyrmex* harvester ants (Wiernasz and Cole 2010), which would be remarkable as it opposes the strong empirical evidence for significant benefits from sperm mixing in *Pogonomyrmex* (Cole and Wiernasz 1999; Wiernasz et al. 2004, 2008). Sperm use has been shown to be random in *Atta* leaf-cutting ants (Holman et al. 2011), but conclusive evidence from other social insects is still limited.

Here we investigated directly whether patriline distributions are consistent over time in *Acromyrmex echinatior*. The queens of this species are obligatorily polyandrous, with all queens investigated so far having sperm from more than one male (range 4–12) in their spermatheca (Sumner et al. 2004; Hughes and Boomsma 2008; Waddington et al. 2010), and have potential life spans of more than 10 years (Weber 1972; log book CSE Copenhagen). To do this, we sampled and genotyped worker cohorts that were either 2 months apart or up to 13 months apart, and compared genotypes of cohorts of eggs laid up to 29 months apart to determine whether there were significant effects of time on the representation of patrilines within subsequent samples from the same colonies.

## Methods

We collected three sets of samples: (1) Workers from nine colonies were sampled twice, with an interval of ca. 2 months (57 days; short-term samples). (2) Workers from four colonies were sampled over four, six, or 13 months to determine whether there might be changes in patriline representation over longer time periods (long-term samples). *A. echinatior* worker life span in laboratory colonies is 6–12 months and new workers are eclosing constantly (Dijkstra and Boomsma 2007), so “long-term” samples were presumed to represent different cohorts. (3) Eggs were sampled from three laboratory colonies at three time points over at least 12 months. Genotypic differences in development rate or mortality could potentially lead to changes in adult patriline frequencies so that, although such effects are not known for social insects, the examination of patriline frequencies in egg batches avoided this potential problem. At each sample point, eggs and workers for the short-term dataset were collected on single day, while workers for the long-term dataset were collected over up to a month. We used a total of 15 monogynous *Acromyrmex echinatior* colonies that were all collected in Gamboa, Republic of Panama between 2000 and 2004 and transported back to Denmark. One colony (Ae 112) was sampled for both the short-term and long-term analyses. Colonies were kept in plastic boxes with the fungus garden under inverted 1000-mL plastic beakers to prevent desiccation, maintained under stable laboratory conditions in climate rooms at 24–26°C and ca. 70% RH, and provided with bramble leaves (*Rubus sp*.), rice, and fresh fruit twice a week.

To compare paternity distributions between cohorts, workers were collected from colonies and stored in 96% ethanol at −20°C until processing. There are two main worker size classes in *Acromyrmex*, small workers (SW) and large workers (LW), and genotype (patriline) may affect the propensity of larvae to develop into these different castes (Weber 1972; Hughes et al. 2003). For the short-term dataset, an equal number of LW (1.8–2.4 mm head width) and SW (0.6–1.2 mm head width) were therefore sampled such that total samples would likely include all patrilines (Hughes and Boomsma 2007), whereas for the long-term dataset we focused on the patrilines represented in the more abundant small workers by collecting individuals with a head width of 1–1.4 mm (Hughes and Boomsma 2004, 2006). All workers were collected from the surface of fungus gardens and were of similar middle-brown coloration and thus approximately of the same physiological age (Armitage and Boomsma 2010). The collection of workers of consistent size, age, and fungus garden location for each data set avoided as much as possible genotypic variation in caste or behavior that could confound the results (Hughes et al. 2003; Hughes and Boomsma 2007; Waddington et al. 2010).

To determine the patriline distribution in egg batches, we collected a small fragment of fungus garden (approximately 1.5 × 1.5 × 1.5 cm) from a colony and checked it under a microscope to ensure it did not contain any eggs, larvae, or pupae. The fungus was then transferred to a petri dish with a moist piece of cotton wool to avoid desiccation, and the queen was located in the colony, collected, and gently placed on the fungus piece. In *A. echinatior,* freshly laid eggs are collected by workers and wrapped in an envelope of fungal hyphae (Armitage et al. 2012) so we additionally transferred five small workers (0.6–1.2 mm head width) to the petri dish along with the queen and left the dish undisturbed in the dark next to the colony in the climate room for 24 h, after which the queen was returned to her colony. Worker reproduction has been shown to be virtually absent in colonies of *A. echinatior* with a live queen (Dijkstra et al. 2005; Dijkstra and Boomsma 2007), so we assumed that eggs laid overnight were all queen derived.

Prior to DNA extraction, the cell division rate of eggs was determined by collecting a subsample of eggs from a batch that had been laid within a two hour window. After approximately 5, 26, 48, and 72 h, some eggs were collected and the number of cells in each of them counted after staining with 5 *μ*L of DAPI working solution (2 *μ*L of a DAPI stock solution [2 mg 4′, 6-diamidino-2-phenylidolindole HCL in 1 ml DMSO]) in 1 mL 0.1 M NaPO_4_ buffer (pH 7.0). DAPI is a stain that binds to DNA and fluoresces blue under ultraviolet light, allowing cell division rates to be estimated and an assessment to be made of sufficient DNA being available for successful extraction and PCR amplification. Cells were counted under an Olympus CX41, EXFO X-Cite 120 fluorescence microscope at 400× magnification. Mean cell number after 47 (±0.7 SE) hours was 606 (±55 SE) cells (Fig. S1), which yielded 24.6 (±4.9 SE) ng *μ*L DNA. After this methodological validation, eggs were henceforth retrieved 2 days after they were laid and transferred to vials containing 96% ethanol and placed in the freezer at −20°C until processing.

We extracted DNA from individual eggs and ant legs using Chelex beads (Bio-Rad, Herlev, Denmark). For eggs, we added 3 *μ*L Proteinase K (Roche diagnostics, Hvidovre, Denmark) for tissue digestion and incubated the samples overnight at 56°C. Genotypes of all samples were determined by amplifying DNA at four highly polymorphic microsatellite loci (*Ech1390*, *Ech3385*, *Ech4126*, and *Ech4225*) (Ortius-Lechner et al. 2000; Hughes and Boomsma 2006). The chance of missing patrilines due to them having identical multilocus genotypes was negligible (estimated nondetection error = 0.00728) (Sumner et al. 2004). PCR reactions were carried out in 10 *μ*L volumes consisting of 1 *μ*L reaction buffer, 4 *μ*L GATC mix, 1 *μ*L forward primer, 1 *μ*L reverse primer, 0.1 *μ*L *Taq* polymerase, 1 *μ*L DNA, and 1.9 *μ*L H_2_O (for eggs we used 2 *μ*L DNA and 0.9 *μ*L H_2_O). DNA was amplified in Hybaid PCR Express, ABI Veriti and ABI 2720 thermal cyclers following published PCR programs (Hughes and Boomsma 2006; Mitchell et al. 2012). PCR products were analyzed on an ABI 3130xl automated sequencer, with allele lengths determined by comparison with the *Liz*500 internal size marker.

Samples were assigned to patrilines based on their paternal alleles. If samples could not be unambiguously assigned to a patriline either because of failure in PCR amplification at a diagnostic locus or because the individual was heterozygous with the same alleles as the queen at all diagnostic loci, they were discarded from the analyses. While unlikely, egg samples could potentially have included haploid eggs, so we also reran the analyses excluding any eggs that were not heterozygous for at least one locus (5, 5, and 12 eggs in colonies Ae150, Ae153 and Ae266, respectively), but this did not change any of the results. We also determined mating frequencies from genotyped worker and egg samples, and calculated effective mating frequencies for each colony based on all samples, following Nielsen et al. 2003—Equ. 16. Patriline skew within each sample and average skew for each colony were calculated using Skew Calculator 2013 (https://www.eeb.ucla.edu/Faculty/Nonacs/PI.html). Two measures of skew were used, Nonacs’ (2000) robust measure of skew “*b*”, and Pamilo and Crozier’s (1996) skew index, called “S_3_” in Skew Calculator, to allow comparison with patriline skew in other polyandrous social insects (Jaffé et al. 2012).

### Statistics

Statistical analyses of differences in paternity representation were carried out using Monte Carlo permutation tests, as implemented in GenoDive 2.0b24 (http://www.bentleydrummer.nl/software/software/GenoDive.html) for Mac OS X. Patrilines were entered as if they were haploid alleles at a single locus, with sampling points entered as populations. Differences in the proportion of different patrilines between sample points were then compared using pairwise and (for the egg and long-term data) overall values of *F*_ST_ as an index of difference in patriline representation, with significance of the differences determined based on 9999 permutations. Values of *F*_ST_ above zero represent greater differences in proportions than expected based on random resampling from the same original distribution, while those below zero represent more similarity in proportions than expected from random sampling. As we performed multiple comparisons within each group (short-term, long-term, and eggs), the *α*-level was adjusted accordingly using sequential Bonferroni corrections.

To provide a measure of the effect sizes for each pairwise comparison within every colony, *Z*-scores (i.e., the number of standard deviations of the observed differentiation above or below the mean permuted value) were calculated from the uncorrected *P*-values. *Z*-scores above zero represent greater differences in proportions than expected based on random resampling from the same original distribution, while those below zero represent higher similarity in proportions than expected from random sampling. For all colonies with more than two samples, we tested whether there was any association between the time interval between samples and change in patriline frequencies within colonies using a Mantel test based on the correlation between the matrix of patriline differences between samples (the effect size matrix from the pairwise differentiation test) and the matrix of the time (in days) between each sample, using 9999 permutations.

To examine whether there were any consistent changes in paternity skew over time, an ANCOVA was conducted comparing the relationship between sampling time and Nonacs’ (2000) robust measure of skew for each of the three (short-term, long-term, and egg) types of samples, with colonies nested within sample types as random variables.

## Results

As expected, egg cell divisions were approximately exponential (Fig. S1), starting within hours after eggs were laid and reaching ca. 500–800 cells after 48 h. All 15 *A. echinatior* colonies were headed by a single, multiply-mated queen, with absolute queen mating frequency (i.e., the number of males fathering offspring; *M*_p_) being 6.07 (±0.51 SE; range 3–9), and genetically effective mating frequency (i.e., the number of males fathering offspring when adjusted for paternity skew; *M*_e,p_) being 3.88 (±0.27) (Table 1). Consistent with this difference, moderate patriline skew was evident and significant for all colonies (Table 1).

**Table 1:** Overview of data and results. Columns display colony ID and data type (WS = workers short term; WL = workers long term), number of sample points, total number of individuals genotyped (*N*), mean number of individuals genotyped per sample (*n* ± SE), actual number of patrilines (*M*_p_), and genetically effective number of patrilines (*M*_e,p_). Result of tests for difference in proportional representation of patrilines (*F*_ST_) before Bonferroni correction, and the sequential Bonferroni corrected level of significance equivalent to *P* = 0.05 are given, as are two measures of paternity skew: *b* (Nonacs 2000) and *S*_3_ (Pamilo and Crozier 1996). Finally, values of the Mantel correlation coefficient (*r*) between *x* and *y*, and its significance are given for those colonies with more than two sample points

Colony	Data type	Sample points	*N*	Sample *n* ± SE	*M*_p_	*M*_e,p_	*F*_ST_	*P*	Bonferroni *α*	Skew (*b*)*	Skew (S_3_)	Mantel *r*	Mantel *P*
112	WS	2	183	91.5 ± 6.5	4	2.7	−0.011	0.970	0.0063	0.113	0.43	–	–
124	WS	2	169	84.5 ± 3.5	7	5.0	−0.001	0.452	0.0063	0.053	0.36	–	–
135	WS	2	167	83.5 ± 1.5	9	5.7	−0.009	0.979	0.0063	0.053	0.41	–	–
219	WS	2	179	89.5 ± 1.5	7	5.0	0.013	0.055	0.0063	0.056	0.35	–	–
220	WS	2	177	88.5 ± 2.5	6	4.5	0.007	0.156	0.0063	0.053	0.33	–	–
221	WS	2	185	92.5 ± 0.5	7	4.1	−0.002	0.492	0.0063	0.093	0.49	–	–
223	WS	2	176	88.0 ± 6.0	7	4.9	0.021	0.019	0.0056	0.065	0.39	–	–
226	WS	2	191	95.5 ± 4.5	3	2.5	−0.007	0.708	0.0063	0.062	0.26	–	–
227	WS	2	180	90.0 ± 1.0	3	2.7	0.014	0.098	0.0063	0.041	0.18	–	–
33	WL	4	782	195.5 ± 31.0	9	4.2	0.000	0.409	0.0167	0.109	0.54	−0.290	0.252
48	WL	3	466	155.3 ± 2.3	8	4.2	0.001	0.259	0.0125	0.113	0.55	0.979	0.170
112	WL	4	470	117.5 ± 4.8	4	2.7	−0.004	0.742	0.0167	0.125	0.46	−0.153	0.581
132	WL	5	497	99.4 ± 10.9	7	4.0	0.002	0.294	0.0167	0.057	0.32	−0.004	0.542
150	Eggs	3	184	61.3 ± 10.2	5	3.0	−0.010	0.839	0.025	0.087	0.38	−0.662	0.332
153	Eggs	3	168	56.0 ± 11.7	4	2.9	−0.004	0.542	0.025	0.073	0.33	−0.025	0.498
266	Eggs	3	168	56.0 ± 4.36	5	2.8	0.006	0.207	0.0167	0.025	0.20	−0.860	0.334

* All values of *b* are significantly greater (*P* < 0.001) than expected under equal division of paternity.

**Figure 1:**
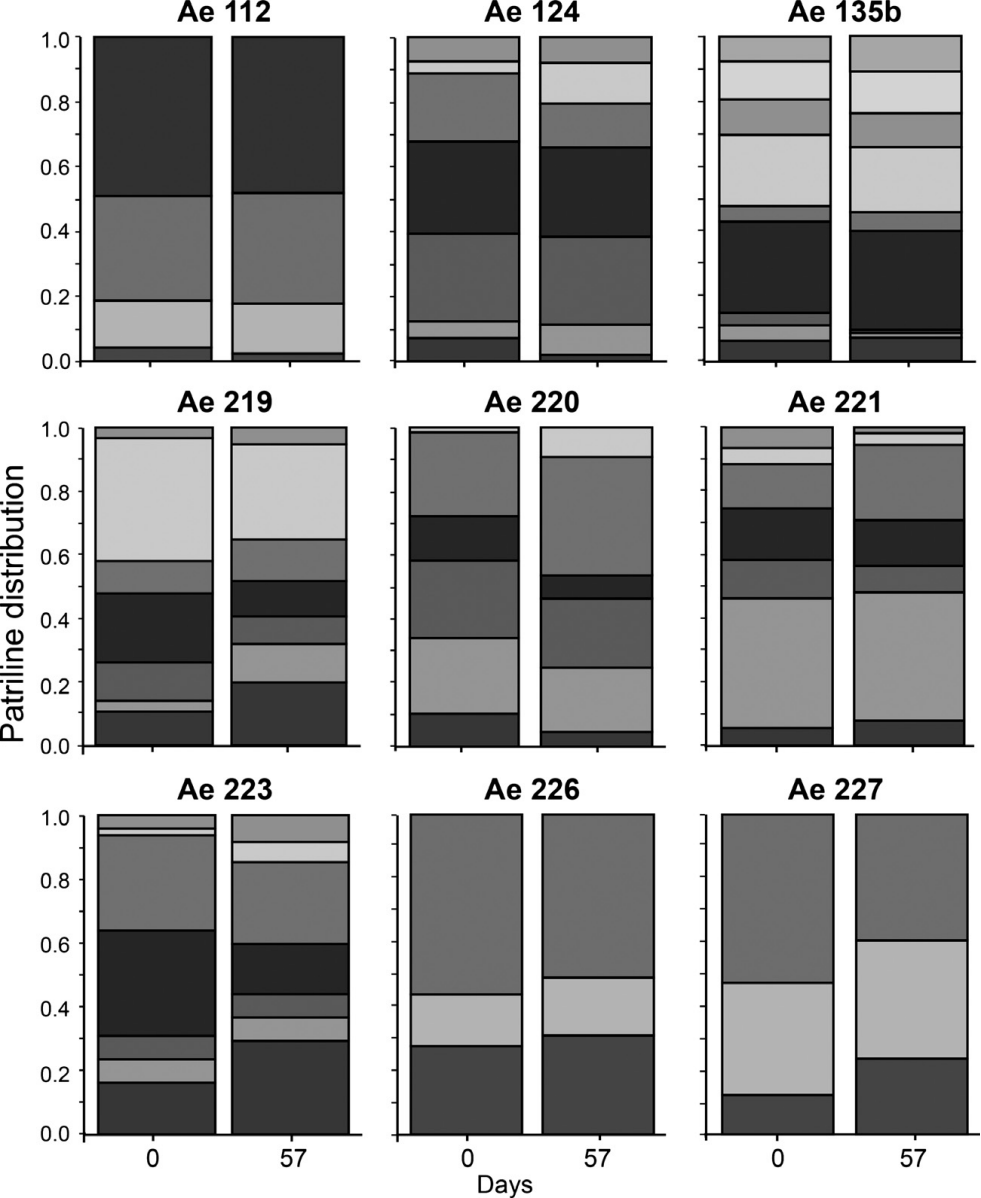
The proportional distribution of patrilines (indicated by different shading) in worker samples from nine colonies of *Acromyrmex echinatior* leaf-cutting ants in the short-term dataset. Samples were collected 57 days apart and consisted of an equal number of large workers and small workers that were all of similar age and were all collected from the surface of fungus gardens.

We found no significant change in the frequency of patrilines among workers in any of the nine colonies across the 57-day interval in the short-term dataset (Fig. 1, Table 1). In one colony (Ae223), the initial analysis showed a potential patriline shift between sample dates, but this was not significant after adjusting the *α*-level to control for multiple comparisons (Fig. 1, Table 1). The pairwise differentiation test of all “short-term” samples showed that the mean difference in patriline representation (effect size) between two samples collected 57 days apart was 0.12 (±0.51 SE), which was not significantly different from zero (*t*_8_ = 0.228, *P* = 0.825) (Fig. 4A).

**Figure 2:**
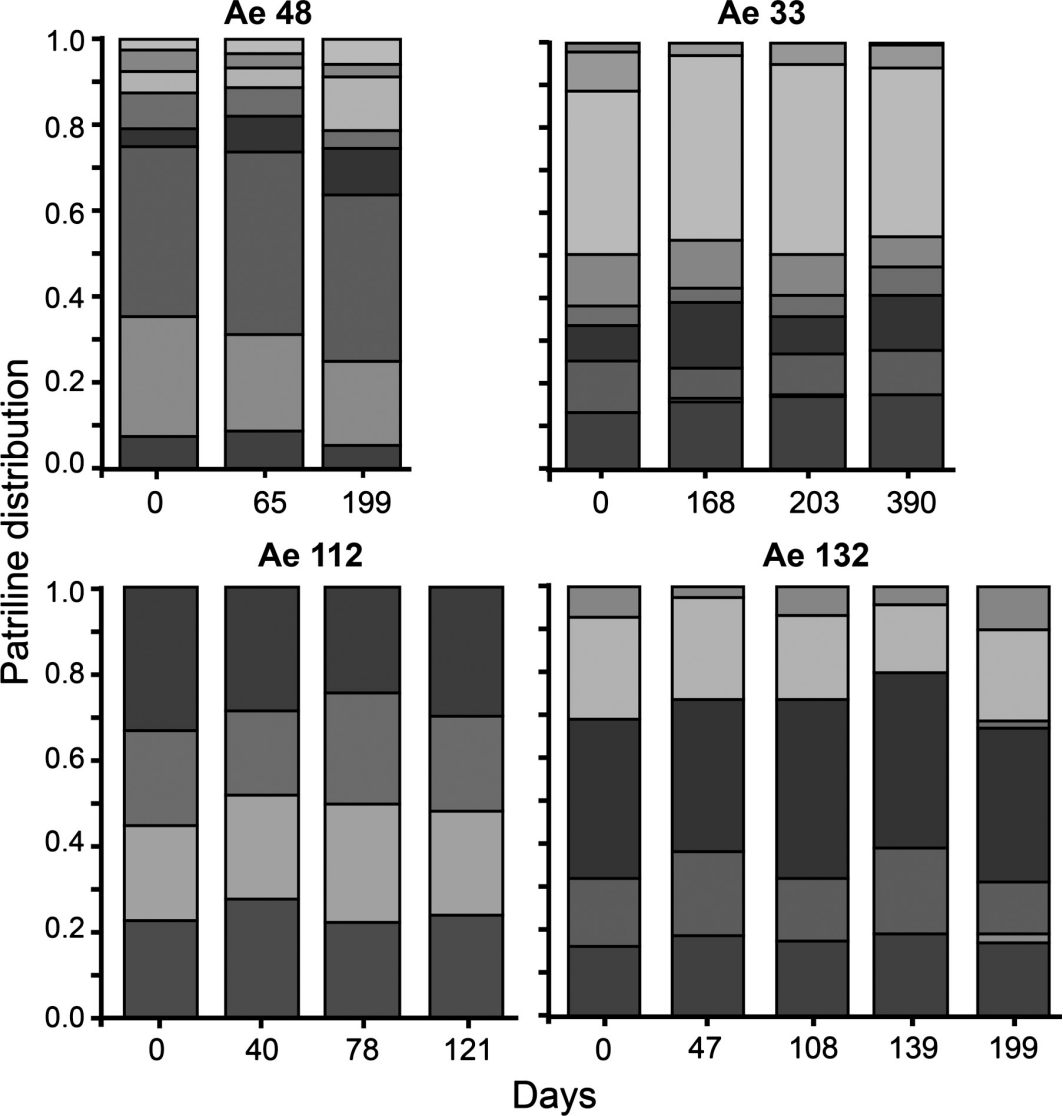
The proportional distribution of patrilines (indicated by different shading) in worker samples from four colonies of *Acromyrmex echinatior* leaf-cutting ants in the long-term dataset. Samples were collected at 3, 4, or 5 time points over 4, 6, or 13 months, depending on the colony. All samples consisted only of small workers (1–1.4 mm head width), that were all of similar age and were all collected from the surface of the fungus garden.

For the long-term dataset we investigated workers from four colonies that were sampled three, four, four, and five times, respectively (Fig. 2). The overall differentiation test showed that none of the samples in any of these colonies had significant changes in patriline representation, even before correcting the *α*-level to adjust for multiple comparisons (Table 1). In the pairwise test of differences in patriline representation between samples for each colony, one colony (Ae48) showed a change in patriline representation (increase in effect size) over time (Fig. 4B), but this was not significant in a Mantel test (*P* = 0.170). All the other colonies also showed deviations across time intervals that were not significantly different from zero (Mantel test, all *P* ≥ 0.252, Table 1).

**Figure 3:**
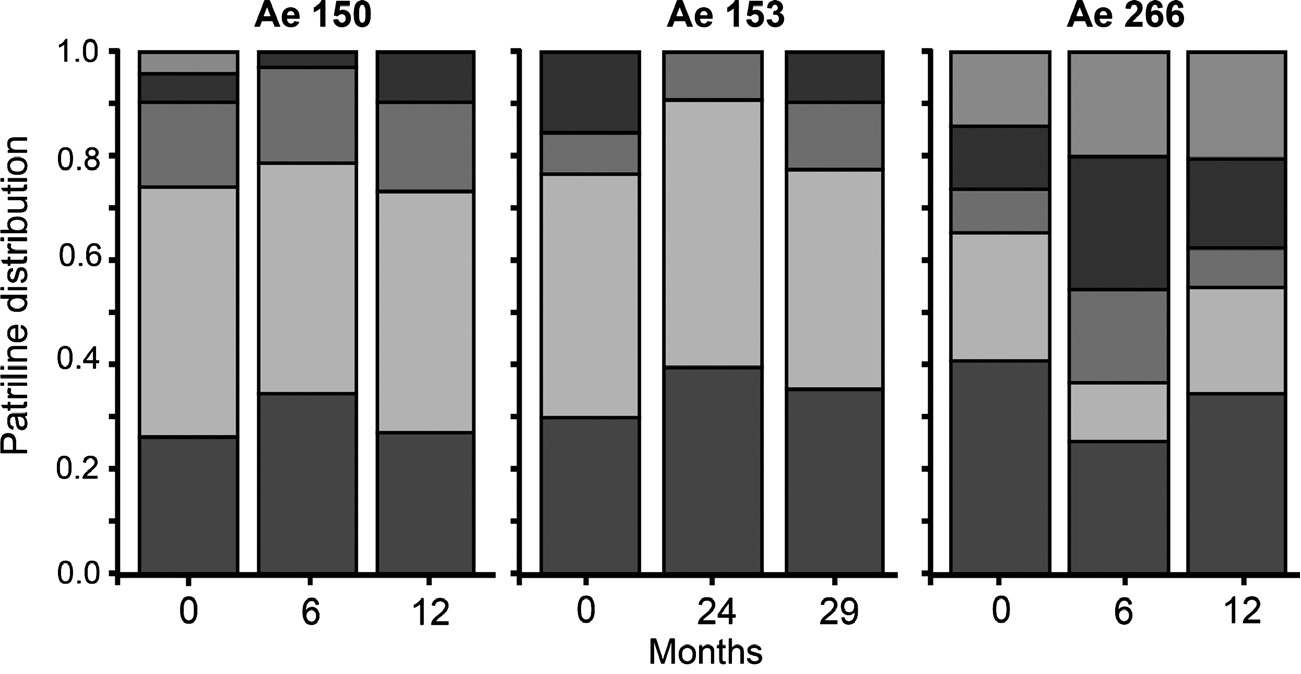
The proportional distribution of patrilines (indicated by different shading) in eggs collected from three colonies of *Acromyrmex echinatior* leaf-cutting ants. Eggs were collected at three different time points in each case, over a 12 or 29 month period depending on the colony.

**Figure 4:**
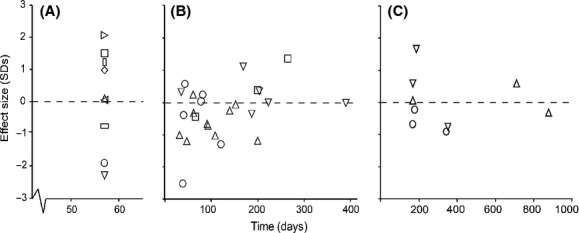
Effect sizes (measured in standard deviation units) as a function of time between samples for *Acromyrmex echinatior* leaf-cutting ant workers sampled from nine colonies over a short time period (A): 57 days; (B): four other colonies over longer time periods of up to 13 months; (C): eggs from three colonies sampled over up to 29 months. Colonies are depicted by different symbols, but the same symbols are used for different colonies across panels (Table 1).

We found no significant variation in patriline distributions across egg samples (Fig. 3; overall differences, all *P* ≥ 0.21). There was also no correlation between the difference in patriline representation among samples and the time between sample points (Mantel test, eggs, all *P* ≥ 0.332). All effect sizes between sample points were within two standard deviations, which is consistent with random differences between samples (Fig. 4C).

Consistent with the lack of changes in patriline representation over time, there was no overall effect of sampling type or length of time between samples on paternity skew (sample type: *F*_2,24_ = 2.21, *P* = 0.131; sample date: *F*_1,24_ = 2.32, *P* = 0.141; sample type × sample date: *F*_2,24_ = 1.35, *P* = 0.277), but there was a significant difference between colonies in their level of skew (*F*_13,24_ = 4.01, *P* = 0.0016).

## Discussion

Our analyses showed that patriline distributions in offspring sampled over both short- and long-time intervals displayed no significant fluctuations, with sperm use from different patrilines remaining consistent across egg batches laid up to more than 2 years apart and across worker cohorts sampled either 2 months apart or repeatedly over a period of up to 1 year. We also found no indications that patriline distributions or skew changed as time intervals between sampling increased. These results are consistent with sperm inside the spermatheca of *A. echinatior* queens being completely mixed and used randomly during egg laying.

The results of our study match the theoretical predictions of sperm mixing in polyandrous social insects, and the empirical evidence for a high degree of sperm mixing in honeybees (Franck et al. 1999, 2002), army ants (Kronauer et al. 2006), eusocial wasps (Goodisman et al. 2007), and *A. colombica* leaf-cutting ants (Holman et al. 2011). The act of mating itself likely incurs costs to queens in terms of increased predation and disease transmission risks (Boomsma and Ratnieks 1996; Crozier and Fjerdingstad 2001; Strassmann 2001; Kraus and Moritz 2010). Storing sperm reduces the immunocompetence of *Atta colombica* leaf-cutting ant queens, with this reduction being more severe when multiple males contribute sperm (Baer et al. 2006). In addition, the evolution of social parasitism in *A. insinuator* has been associated with an almost complete reversion to monandry, implying that the costs of polyandry are significant enough to be selected against when worker genetic diversity is provided by a host colony (Sumner et al. 2004). High and apparently obligate polyandry in *A. echinatior* queens must therefore provide significant benefits to have evolved and be maintained, most likely because it increases colony performance due to improved division of labor (Hughes et al. 2003; Smith et al. 2008; Waddington et al. 2010; Constant et al. 2012) or disease resistance (Hughes and Boomsma 2004, 2006; Hughes et al. 2010), and such benefits would only be maximized if sperm was completely mixed after storage.

Our present results contrast with a recent study arguing that sperm clumping occurs in *Pogonomyrmex occidentalis* harvester ants (Wiernasz and Cole 2010). Selective benefits of genetically diverse colonies have been documented in *Pogonomyrmex* (Cole and Wiernasz 1999; Wiernasz et al. 2004; Lubertazzi et al. 2013), and sperm clumping is therefore expected to be harmful to the overall colony interests in these ants. While sperm clumping has been documented in *F. truncorum*, this ant is not directly comparable because polyandry is facultative and workers respond to colony-level variation in relatedness asymmetry induced by single mating versus multiple mating by biasing sex allocation in opposite directions as predicted by inclusive fitness theory (Sundström and Boomsma 2000). Queens of both *P. occidentalis* and *A. echinatior*, in contrast, are highly polyandrous, a difference in mating system that is not merely part of a continuum, but fundamental (Boomsma and Ratnieks 1996; Hughes et al. 2008; Boomsma 2013), and in neither species do workers split sex ratios in response to variation in effective queen mating frequency (Dijkstra and Boomsma 2008; Wiernasz and Cole 2009). While it is possible that unknown factors in the biology of *P. occidentalis* could make sperm clumping less disruptive to colony fitness, the strong evidence for benefits of worker genetic diversity in *Pogonomyrmex* (Cole and Wiernasz 1999; Wiernasz et al. 2004) makes it seem more likely that their queens too would gain the highest possible fitness through complete sperm admixture.

Although we find consistency in patriline distributions between samples, the proportion of different patrilines within each colony was noticeably skewed (Figs 1–3, Table 1), with some males siring more than half of the offspring and other males having very low paternity shares. Levels of paternity skew (*S*_3_) in *A. echinatior* (Mean ± SE: 0.37 ± 0.03) were similar to those recorded for other highly polyandrous (Mean No. fathers >2) ants in general (0.30 ± 0.05) and those specifically in the subfamily Myrmicinae (0.30 ± 0.05) (Jaffé et al. 2012). While this could simply represent differences in ejaculate size between males, it might also be the result of sperm competition or cryptic female choice (Eberhard and Cordero 1995; Simmons 2005). Shortly after insemination, multiple ejaculates overlap in the female’s reproductive tract before they reach the final sperm storage organ, and while direct observations from *A. echinatior* are lacking, data on the congeneric species *Acromyrmex versicolor* show that an order of magnitude more sperm is deposited in the reproductive tract than is finally stored in the spermatheca (Reichardt and Wheeler 1996). This suggests that sperm cells might initially compete for storage, a process that has been documented for *A. echinatior* in terms of male accessory gland secretion being more supportive to own sperm than to alien sperm (Den Boer et al. 2010). In *Atta colombica*, it has also been shown that secretions from the queen spermatheca terminate any such hostility between ejaculates, but whether this also applies to *A. echinatior* remains unknown.

The number of stored sperm in eusocial insect queens will ultimately limit reproductive success and colony longevity (Cole 1983; Boomsma and Ratnieks 1996; Den Boer et al. 2009), so we expect queens to use all the sperm they stored in their spermatheca according to a “fair raffle” principle (Parker 1990), rather than excluding the sperm of certain males as occurs in many noneusocial polyandrous taxa where females only store sperm until they remate. A recent study on the leaf-cutting ant *Atta colombica* confirmed this fair raffle principle of sperm use (Holman et al. 2011). Yet, while equal paternity across all fathers siring offspring would maximize colony genetic diversity benefits from multiple mating to queens (it would make the genetically effective mating frequency equal to the numerical mating frequency), variation in ejaculate size or quality, or in its success during the initial antagonistic competition in the female reproductive tract may produce paternity skew that queens cannot control (although they may have some influence on sperm competition; Jaffé et al. 2012). When males do not have specific interests in opposing the breakup of their ejaculates during and after final sperm storage, there will be no selection on them to resist sperm mixing. This is different from *F. truncorum* in which split sex ratio idiosyncrasies imply that stored ejaculates have a joint interest in maintaining their coherence (Sundström and Ratnieks 1998; Sundström and Boomsma 2000).

Our results show that sperm clumping does not offer an alternative explanation for the differential representation of patrilines across different castes in *A. echinatior* (Hughes et al. 2003; Hughes and Boomsma 2008) as suggested by Wiernasz and Cole (2010). A genetic predisposition for certain genotypes to develop into certain castes is of considerable importance in eusocial insects as any male carrying genes that increase the chance that his daughters develop into gynes rather than workers will effectively parasitize on colony resources by producing more than his fair share of sexual offspring. Evidence for such selfish patrilines has been found for replacement queen rearing in honeybees (Moritz et al. 2005), emergency queen rearing through thelytokous parthenogenesis by workers in *Cataglyphis cursor* ants (Chêron et al. 2011), and in gyne production in *A. echinatior* leaf-cutting ants (Hughes and Boomsma 2008). While Wiernasz and Cole (2010) argued that sperm clumping might be more common than previously considered, the results of our present study make it unlikely that genotypic variation in caste propensity (Hughes et al. 2003; Hughes and Boomsma 2008) can be explained by deviations from random use of stored sperm.

Our results match findings in previous studies on honeybees, army ants, and leaf-cutting ants (Franck et al. 1999; Kronauer et al. 2006; Holman et al. 2011; Jaffé et al. 2012) and suggest that sperm mixing is likely to be a general phenomenon in eusocial insects with obligate, highly multiple mating, because it maximizes the benefits gained from genetically diverse colonies. The logic of complete sperm mixing is compelling because it makes a direct connection between the costs of polyandry on one hand and the benefits of higher genetic diversity for division of labor and/or disease resistance in offspring on the other. This connection has gained consistent support in recent years (Kraus and Page 1998; Cole and Wiernasz 1999; Tarpy 2003; Hughes and Boomsma 2004, 2006) for eusocial insects with obligate, highly multiple mating. Neither sex remates later in life so their lifetime-committed interests in colony survival and productivity during the ergonomic stage of colony growth (several years of exclusive worker production in ants) before first reproduction are fully aligned (Boomsma et al. 2005a). Sperm clumping would reduce a major selective advantage of polyandry for colony fitness and therefore decrease the fitness of both queens and their multiple male partners, making it likely to be selected against in the eusocial insects where all queens mate with multiple males.

## Supporting Information

Data S1 Patriline summary table. Nest ID, Sample type (workers short-/long term, eggs), sample date, patriline ID, number of individuals assigned to each patriline.

Figure S1 The number of cells in eggs of *Acromyrmex echinatior* leaf-cutting ants as a function of time after laying.
