# Supplementary material for: Sperm mixing in the polyandrous leaf-cutting ant Acromyrmex echinatior
Source: Ecol Evol. 2014 Sep 2;4(18):3571–82. doi: 10.1002/ece3.1176 (PMC4224532; doi:10.1002/ece3.1176)
Supplement: Data S1 — Patriline summary table. Nest ID, Sample type (workers short-/long term, eggs), sample date, patriline ID, number of individuals assigned to each patriline. [file ece30004-3571-sd1.pdf]

Nest	Type	Sample	Patriline	No individuals	Sum
Ae 112	Long	15/09/02	1	2	
Ae 112	Long	15/09/02	2	17	
Ae 112	Long	15/09/02	3	46	
Ae 112	Long	15/09/02	4	45	
Ae 112	Long	25/10/02	1	2	
Ae 112	Long	25/10/02	2	15	
Ae 112	Long	25/10/02	3	57	
Ae 112	Long	25/10/02	4	46	
Ae 112	Long	02/12/02	1	2	
Ae 112	Long	02/12/02	2	12	
Ae 112	Long	02/12/02	3	54	
Ae 112	Long	02/12/02	4	42	
Ae 112	Long	14/01/03	1	3	
Ae 112	Long	14/01/03	2	18	
Ae 112	Long	14/01/03	3	52	
Ae 112	Long	14/01/03	4	57	470
Ae 112	Short	18/09/03	1	4	
Ae 112	Short	18/09/03	2	14	
Ae 112	Short	18/09/03	3	32	
Ae 112	Short	18/09/03	4	48	
Ae 112	Short	14/11/03	1	2	
Ae 112	Short	14/11/03	2	13	
Ae 112	Short	14/11/03	3	29	
Ae 112	Short	14/11/03	4	41	183
Ae124	Short	18/09/03	1	6	
Ae124	Short	18/09/03	2	4	
Ae124	Short	18/09/03	3	22	
Ae124	Short	18/09/03	4	23	
Ae124	Short	18/09/03	5	17	
Ae124	Short	18/09/03	6	3	
Ae124	Short	18/09/03	7	6	
Ae124	Short	14/11/03	1	2	
Ae124	Short	14/11/03	2	8	
Ae124	Short	14/11/03	3	24	
Ae124	Short	14/11/03	4	24	
Ae124	Short	14/11/03	5	12	
Ae124	Short	14/11/03	6	11	
Ae124	Short	14/11/03	7	7	169

Nest	Type	Sample	Patriline	No individuals	Sum
Ae132	Long	29/06/02	1	18	
Ae132	Long	29/06/02	3	18	
Ae132	Long	29/06/02	4	42	
Ae132	Long	29/06/02	8	27	
Ae132	Long	29/06/02	9	8	
Ae132	Long	15/08/02	1	14	
Ae132	Long	15/08/02	3	15	
Ae132	Long	15/08/02	4	27	
Ae132	Long	15/08/02	8	18	
Ae132	Long	15/08/02	9	2	
Ae132	Long	15/10/02	1	18	
Ae132	Long	15/10/02	3	15	
Ae132	Long	15/10/02	4	43	
Ae132	Long	15/10/02	8	20	
Ae132	Long	15/10/02	9	7	
Ae132	Long	15/11/02	1	14	
Ae132	Long	15/11/02	3	15	
Ae132	Long	15/11/02	4	30	
Ae132	Long	15/11/02	8	12	
Ae132	Long	15/11/02	9	3	
Ae132	Long	14/01/03	1	22	
Ae132	Long	14/01/03	2	3	
Ae132	Long	14/01/03	3	16	
Ae132	Long	14/01/03	4	47	
Ae132	Long	14/01/03	7	2	
Ae132	Long	14/01/03	8	28	
Ae132	Long	14/01/03	9	13	497
Ae135B	Short	18/09/03	1	5	
Ae135B	Short	18/09/03	2	4	
Ae135B	Short	18/09/03	3	3	
Ae135B	Short	18/09/03	4	23	
Ae135B	Short	18/09/03	5	4	
Ae135B	Short	18/09/03	6	18	
Ae135B	Short	18/09/03	7	9	
Ae135B	Short	18/09/03	8	10	
Ae135B	Short	18/09/03	9	6	
Ae135B	Short	14/11/03	1	6	
Ae135B	Short	14/11/03	2	1	
Ae135B	Short	14/11/03	3	1	
Ae135B	Short	14/11/03	4	26	
Ae135B	Short	14/11/03	5	5	
Ae135B	Short	14/11/03	6	17	
Ae135B	Short	14/11/03	7	9	
Ae135B	Short	14/11/03	8	11	
Ae135B	Short	14/11/03	9	9	167

Nest	Type	Sample	Patriline	No individuals	Sum
Ae150	Eggs	29/07/10	1	18	
Ae150	Eggs	29/07/10	2	34	
Ae150	Eggs	29/07/10	3	12	
Ae150	Eggs	29/07/10	4	4	
Ae150	Eggs	29/07/10	5	2	
Ae150	Eggs	21/01/11	1	24	
Ae150	Eggs	21/01/11	2	31	
Ae150	Eggs	21/01/11	3	12	
Ae150	Eggs	21/01/11	4	2	
Ae150	Eggs	07/07/11	1	13	
Ae150	Eggs	07/07/11	2	19	
Ae150	Eggs	07/07/11	3	9	
Ae150	Eggs	07/07/11	4	4	184
Ae153	Eggs	10/02/09	1	19	
Ae153	Eggs	10/02/09	2	30	
Ae153	Eggs	10/02/09	3	5	
Ae153	Eggs	10/02/09	4	10	
Ae153	Eggs	21/01/11	1	13	
Ae153	Eggs	21/01/11	2	17	
Ae153	Eggs	21/01/11	3	3	
Ae153	Eggs	07/07/11	1	25	
Ae153	Eggs	07/07/11	2	30	
Ae153	Eggs	07/07/11	3	9	
Ae153	Eggs	07/07/11	4	7	168
Ae219	Short	18/09/03	1	9	
Ae219	Short	18/09/03	2	3	
Ae219	Short	18/09/03	3	11	
Ae219	Short	18/09/03	4	19	
Ae219	Short	18/09/03	5	9	
Ae219	Short	18/09/03	6	34	
Ae219	Short	18/09/03	7	3	
Ae219	Short	14/11/03	1	18	
Ae219	Short	14/11/03	2	11	
Ae219	Short	14/11/03	3	8	
Ae219	Short	14/11/03	4	10	
Ae219	Short	14/11/03	5	12	
Ae219	Short	14/11/03	6	27	
Ae219	Short	14/11/03	7	5	179
Ae220	Short	18/09/03	1	9	
Ae220	Short	18/09/03	2	22	
Ae220	Short	18/09/03	3	22	
Ae220	Short	18/09/03	4	13	
Ae220	Short	18/09/03	5	24	
Ae220	Short	18/09/03	6	1	
Ae220	Short	14/11/03	1	4	
Ae220	Short	14/11/03	2	17	
Ae220	Short	14/11/03	3	19	
Ae220	Short	14/11/03	4	6	
Ae220	Short	14/11/03	5	32	
Ae220	Short	14/11/03	6	8	177

Nest	Type	Sample	Patriline	No individuals	Sum
Ae221	Short	18/09/03	1	5	
Ae221	Short	18/09/03	2	38	
Ae221	Short	18/09/03	3	11	
Ae221	Short	18/09/03	4	15	
Ae221	Short	18/09/03	5	13	
Ae221	Short	18/09/03	6	5	
Ae221	Short	18/09/03	7	6	
Ae221	Short	14/11/03	1	7	
Ae221	Short	14/11/03	2	37	
Ae221	Short	14/11/03	3	8	
Ae221	Short	14/11/03	4	13	
Ae221	Short	14/11/03	5	22	
Ae221	Short	14/11/03	6	3	
Ae221	Short	14/11/03	7	2	185
Ae223	Short	18/09/03	1	15	
Ae223	Short	18/09/03	2	7	
Ae223	Short	18/09/03	3	7	
Ae223	Short	18/09/03	4	31	
Ae223	Short	18/09/03	5	28	
Ae223	Short	18/09/03	6	2	
Ae223	Short	18/09/03	7	4	
Ae223	Short	14/11/03	1	24	
Ae223	Short	14/11/03	2	6	
Ae223	Short	14/11/03	3	6	
Ae223	Short	14/11/03	4	13	
Ae223	Short	14/11/03	5	21	
Ae223	Short	14/11/03	6	5	
Ae223	Short	14/11/03	7	7	176
Ae226	Short	18/09/03	1	25	
Ae226	Short	18/09/03	2	15	
Ae226	Short	18/09/03	3	51	
Ae226	Short	14/11/03	1	31	
Ae226	Short	14/11/03	2	18	
Ae226	Short	14/11/03	3	51	191
Ae227	Short	18/09/03	1	11	
Ae227	Short	18/09/03	2	32	
Ae227	Short	18/09/03	3	48	
Ae227	Short	14/11/03	1	21	
Ae227	Short	14/11/03	2	33	
Ae227	Short	14/11/03	3	35	180
Ae266	Eggs	21/07/10	21	20	
Ae266	Eggs	21/07/10	22	12	
Ae266	Eggs	21/07/10	23	4	
Ae266	Eggs	21/07/10	24	6	
Ae266	Eggs	21/07/10	25	7	
Ae266	Eggs	21/01/11	21	14	
Ae266	Eggs	21/01/11	22	6	
Ae266	Eggs	21/01/11	23	10	
Ae266	Eggs	21/01/11	24	14	
Ae266	Eggs	21/01/11	25	11	
Ae266	Eggs	07/07/11	21	22	
Ae266	Eggs	07/07/11	22	13	
Ae266	Eggs	07/07/11	23	5	
Ae266	Eggs	07/07/11	24	11	
Ae266	Eggs	07/07/11	25	13	168

Nest	Type	Sample	Patriline	No individuals	Sum
Ae33	Long	20/12/01	1	17	
Ae33	Long	20/12/01	3	16	
Ae33	Long	20/12/01	4	11	
Ae33	Long	20/12/01	5	6	
Ae33	Long	20/12/01	7	16	
Ae33	Long	20/12/01	8	50	
Ae33	Long	20/12/01	9	12	
Ae33	Long	20/12/01	10	3	
Ae33	Long	06/06/02	1	38	
Ae33	Long	06/06/02	2	2	
Ae33	Long	06/06/02	3	18	
Ae33	Long	06/06/02	4	37	
Ae33	Long	06/06/02	5	8	
Ae33	Long	06/06/02	7	28	
Ae33	Long	06/06/02	8	106	
Ae33	Long	06/06/02	9	7	
Ae33	Long	11/07/02	1	43	
Ae33	Long	11/07/02	2	1	
Ae33	Long	11/07/02	3	24	
Ae33	Long	11/07/02	4	22	
Ae33	Long	11/07/02	5	13	
Ae33	Long	11/07/02	7	24	
Ae33	Long	11/07/02	8	113	
Ae33	Long	11/07/02	9	13	
Ae33	Long	14/01/03	1	27	
Ae33	Long	14/01/03	3	16	
Ae33	Long	14/01/03	4	20	
Ae33	Long	14/01/03	5	10	
Ae33	Long	14/01/03	7	11	
Ae33	Long	14/01/03	8	61	
Ae33	Long	14/01/03	9	8	
Ae33	Long	14/01/03	10	1	782
Ae48	Long	25/04/02	1	12	
Ae48	Long	25/04/02	2	44	
Ae48	Long	25/04/02	3	63	
Ae48	Long	25/04/02	4	7	
Ae48	Long	25/04/02	5	13	
Ae48	Long	25/04/02	6	8	
Ae48	Long	25/04/02	7	8	
Ae48	Long	25/04/02	8	4	
Ae48	Long	29/06/02	1	13	
Ae48	Long	29/06/02	2	34	
Ae48	Long	29/06/02	3	64	
Ae48	Long	29/06/02	4	13	
Ae48	Long	29/06/02	5	10	
Ae48	Long	29/06/02	6	7	
Ae48	Long	29/06/02	7	5	
Ae48	Long	29/06/02	8	5	
Ae48	Long	14/01/03	1	8	
Ae48	Long	14/01/03	2	31	
Ae48	Long	14/01/03	3	60	
Ae48	Long	14/01/03	4	17	
Ae48	Long	14/01/03	5	7	
Ae48	Long	14/01/03	6	19	
Ae48	Long	14/01/03	7	5	
Ae48	Long	14/01/03	8	9	466
