# Supplementary figures and images for: Sperm mixing in the polyandrous leaf-cutting ant Acromyrmex echinatior
Source: Ecol Evol. 2014 Sep 2;4(18):3571–82. doi: 10.1002/ece3.1176 (PMC4224532; doi:10.1002/ece3.1176)

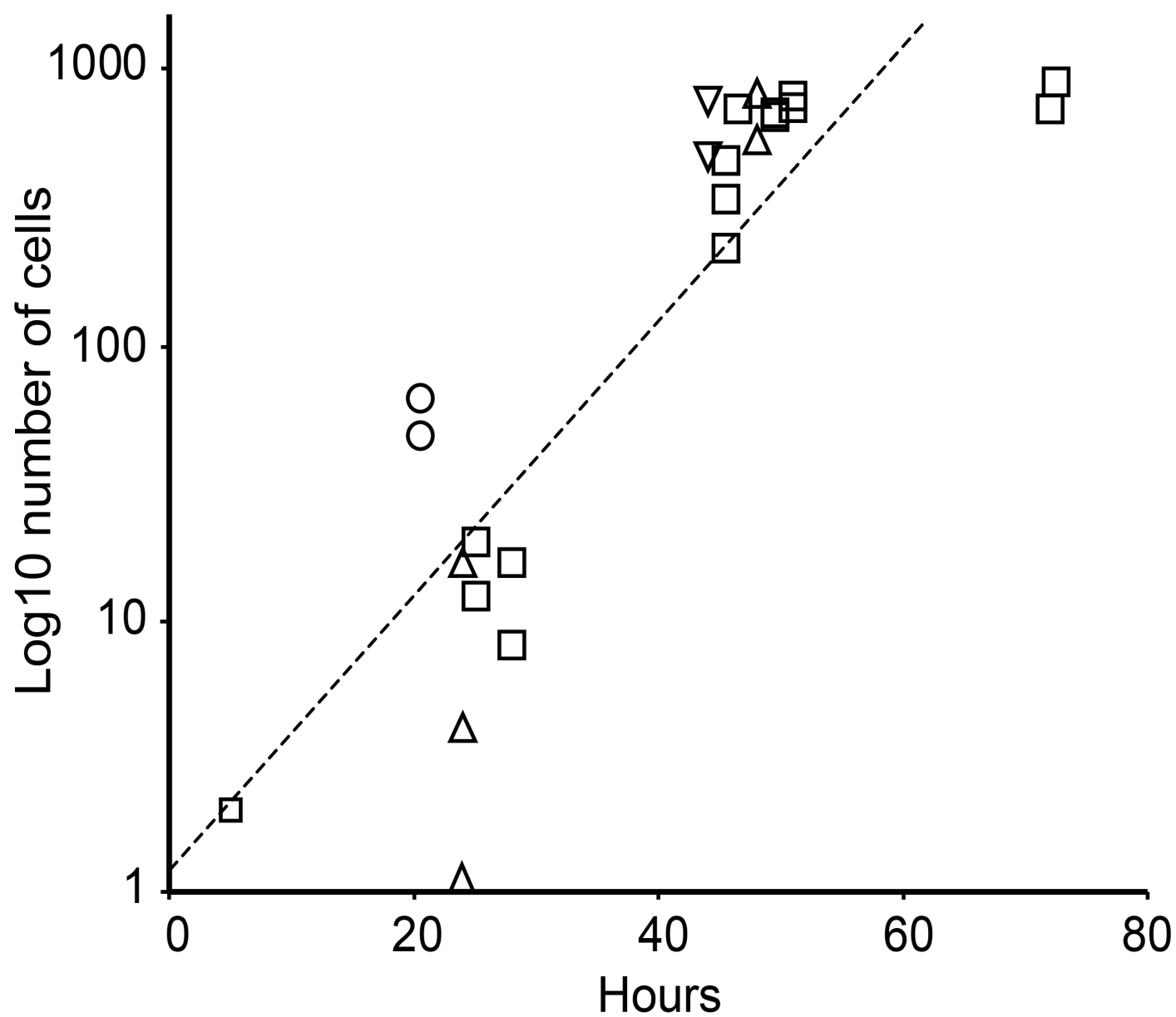

Supplement: Figure S1 — The number of cells in eggs of Acromyrmex echinatior leaf-cutting ants as a function of time after laying. [file ece30004-3571-sd2.pdf]
